# Between moral judgment and randomness: existential anxiety, meaning-making, and the pathway from cognition to health

**DOI:** 10.3389/fpsyg.2026.1845971

**Published:** 2026-06-25

**Authors:** Bandara Bandaranayake

**Affiliations:** Evolving Mindz, Melbourne, VIC, Australia

**Keywords:** death anxiety, existential anxiety, intolerance of uncertainty, meaning-making, psychoneuroimmunology, religion and coping, stress regulation, Terror Management Theory

## Abstract

Human beings possess a distinctive psychological capacity to anticipate their own mortality and to interpret suffering within broader frameworks of meaning. This capacity gives rise to existential anxiety, particularly in contexts of illness, aging, loss, and uncertainty. While existential anxiety has been widely examined in philosophy and psychology, comparatively less attention has been given to the interpretive frameworks through which individuals explain suffering in clinical settings and to the biological consequences of those interpretations. This conceptual paper introduces the *Randomness Intolerance Hypothesis*, which proposes that individuals have limited psychological tolerance for interpreting significant life events as random and therefore construct explanatory frameworks that impose moral or cosmic order. Drawing on existential psychology, terror management theory, and the cognitive science of religion, the paper identifies three primary interpretive pathways: randomness, moral causation, and cosmic purpose. These pathways are illustrated through clinical case examples. The paper further proposes a *Meaning → Biology → Health pathway,* suggesting that meaning-making processes influence emotional states, stress physiology, and long-term health outcomes. Interpretations that reduce perceived threat may support adaptive regulation, whereas those that sustain fear, guilt, or helplessness may contribute to chronic dysregulation. The paper concludes with implications for psychotherapy and future empirical research.

## 1 Introduction

Human beings possess a remarkable psychological capacity: the ability to imagine their own death. This capacity enables reflection on mortality, the fairness of life, and the inevitability of suffering and loss. While such reflection has contributed to philosophy, religion, morality, and culture, it also entails a distinctive form of psychological distress—existential anxiety.

Unlike situational fear, which arises in response to immediate threats, existential anxiety concerns the fundamental conditions of human existence, including awareness of death, uncertainty about meaning, limits of control, and the possibility that suffering may lack ultimate explanation. Existential thinkers and therapists have long argued that anxiety is not merely a pathological symptom but an inevitable consequence of human freedom, self-awareness, responsibility, and mortality (Binswanger, 1963; Boss, 1979; May, 1950, 1983; Yalom, 1980). These concerns become particularly salient in the context of illness, aging, bereavement, and cumulative life adversity.

This paper makes three contributions. First, it introduces the *Randomness Intolerance Hypothesis*, proposing that individuals have limited tolerance for interpreting major life events as random. Second, it integrates existential psychology with cognitive and evolutionary perspectives on meaning-making. Third, it proposes a *Meaning → Biology → Health pathway*, linking interpretive processes to physiological regulation and health outcomes.

## 2 Existential anxiety, death awareness, and meaning

Existential psychology emphasizes that anxiety is not inherently pathological. Some forms of anxiety arise directly from the conditions of human existence. Yalom (1980) identified four ultimate concerns—death, freedom, isolation, and meaninglessness—arguing that awareness of mortality occupies a central position because it shapes all human striving. Knowledge of finitude raises fundamental questions regarding responsibility, purpose, and the value of action.

Frankl (1984), drawing on his experiences in Nazi concentration camps, emphasized that human beings can endure extraordinary suffering when it can be situated within a meaningful framework. He proposed that individuals are primarily motivated by a “will to meaning,” and that psychological distress intensifies when suffering is experienced as meaningless.

These existential perspectives converge with the work of Becker (1973), who argued that awareness of mortality creates a fundamental existential problem that human beings manage through cultural systems of meaning, identity, and symbolic immortality. Building on these ideas, Terror Management Theory (TMT) proposes that awareness of mortality creates the potential for overwhelming psychological terror (Greenberg et al., 1997; Pyszczynski et al., 2015). According to TMT, cultural worldviews buffer this terror by providing systems of meaning that offer symbolic immortality and standards of self-worth. In this sense, TMT may be understood as an empirical elaboration of many of Becker’s central propositions concerning mortality awareness and meaning-making.

### 2.1 Moral meaning and human interpretation

Humans appear to seek not only causal explanations but also moral explanations for significant life events. Across cultures, individuals frequently ask not merely “Why did this happen?” but also “Why did this happen to me?” and “Was it deserved?” Such questions transform suffering from a purely causal problem into a moral one. Perceptions of fairness, responsibility, justice, and accountability appear deeply embedded in human social cognition and may influence how individuals interpret adversity. Consequently, existential anxiety often activates not only a search for meaning but also a search for moral order.

Cultural worldviews, including religious belief systems, serve two critical functions: they explain suffering and death, and they allow individuals to perceive themselves as participants in a meaningful order that transcends individual mortality.

Religious narratives such as divine justice, karma, providence, and destiny are particularly effective in transforming apparently random events into morally structured explanations.

Across cultures, suffering is rarely left uninterpreted. Illness may be understood as punishment, test, destiny, karmic consequence, or spiritual opportunity, or an inherent aspect of biological existence. Even in secular contexts, suffering is frequently understood as psychologically meaningful or developmentally transformative. Winnicott (1965) argued that manageable frustration and disappointment may contribute to psychological growth, autonomy, and resilience. Meaning-making therefore involves not only explaining suffering but also integrating experiences that may contribute to maturation and adaptation. Through such narratives, individuals attempt to answer a psychologically urgent question: *How can I live with what has happened?*

## 3 Aging, vulnerability, and the intensification of existential concerns

Existential anxiety may be understood as a fundamental condition of human existence arising from mortality, vulnerability, uncertainty, and the limits of control. Although present throughout the lifespan, these concerns often become more salient in later adulthood. Aging introduces repeated reminders of mortality and bodily vulnerability, while physical decline, chronic illness, and reduced independence challenge assumptions of continuity and control. Social networks may contract as peers die, and the future becomes increasingly finite. However, experiences of vulnerability are not unique to aging; dependency, uncertainty, loss, and bodily fragility are encountered across the lifespan, beginning in infancy and continuing throughout development. Later life may therefore be understood not as the origin of existential anxiety but as a period in which existential concerns become more difficult to avoid or defer.

These changes leave individuals with fewer psychological mechanisms through which to distance themselves from thoughts of death. Mortality becomes less abstract and more immediate, increasing existential salience. Later life is also characterised by life review, a process through which individuals reflect on past experiences, regrets, losses, and accomplishments (Butler, 1963). While life review may foster integration and acceptance, it may also re-activate unresolved grief, guilt, and relational wounds.

Importantly, aging often heightens sensitivity to *uncertainty and perceived lack of control,* both of which are central to existential anxiety. Physical decline, illness, bereavement, and increasing awareness of mortality may intensify existential concerns for some individuals. Questions of fairness and justice become more salient: Why did certain hardships occur? Why do some suffer more than others? Why do some lives end peacefully while others involve prolonged decline? When such experiences cannot be integrated into a coherent interpretive framework, psychological distress may intensify.

At the same time, later adulthood is not uniformly associated with increased existential distress. For many individuals, aging is accompanied by greater acceptance, emotional maturity, wisdom, and life integration. Whether existential concerns intensify or become more fully integrated may depend on personal, relational, cultural, and spiritual resources. From the perspective of the present paper, these conditions increase the likelihood that individuals will seek structured explanations to restore coherence. In this sense, later life may represent a developmental context in which the *intolerance of randomness becomes particularly pronounced,* as individuals confront the limits of control, predictability, and meaning.

The clinical illustrations below demonstrate how individuals from different cultural and religious backgrounds attempt to resolve these tensions through distinct meaning-making systems.

## 4 Evolutionary and cognitive foundations of meaning-making

### 4.1 Cognitive architecture: agency detection, pattern detection, and inferential bias

Human beings do not confront uncertainty with a neutral mind. Research in the cognitive science of religion suggests that the human mind is predisposed to detect agency, infer intention, and search for causal patterns, particularly under conditions of ambiguity or threat (Atran, 2002; Barrett, 2012; Boyer, 2001). A central concept in this literature is the Hypersensitive Agency Detection Device (HADD), which refers to a cognitive bias toward perceiving agents behind uncertain events. From an evolutionary standpoint, mistaking a non-agent for an agent may be less costly than failing to detect a predator, rival, or hostile other.

Related biases toward pattern detection and teleological reasoning make humans inclined to ask not only what happened but why it happened and for what purpose. Research suggests that people often seek explanations that transform uncertainty into intelligible narratives, particularly during periods of threat, loss, or adversity (Atran, 2002; Boyer, 2001). These cognitive tendencies do not in themselves produce religion, but they create a psychological environment in which intentional, moral, and supernatural explanations can readily emerge.

Importantly, agency detection may generate more than simple causal explanations. Once events are interpreted as resulting from intentional agents, questions concerning responsibility, justice, fairness, and accountability naturally arise. Human beings appear predisposed not only to search for causes but also to search for meaning within social and moral frameworks. Consequently, existential anxiety may activate a search for both explanatory and moral order, helping to explain why suffering is frequently interpreted through narratives of karma, divine judgment, destiny, providence, or moral consequence rather than purely mechanistic explanations.

### 4.2 Minimally counterintuitive beliefs and cultural transmission

While evolved cognitive biases may create receptivity to supernatural ideas, they do not explain why particular religious concepts spread so successfully across cultures and generations. Boyer (2001) proposed that many religious ideas possess a distinctive cognitive structure known as minimally counterintuitive beliefs. Such beliefs violate some ordinary expectations about the world while preserving enough familiar characteristics to remain understandable and memorable.

For example, an invisible being who possesses thoughts, intentions, emotions, and moral concerns violates expectations regarding physical presence while remaining cognitively comprehensible because it retains familiar human-like psychological characteristics. Similarly, concepts such as ancestral spirits, gods, angels, demons, or karmic forces introduce supernatural elements without abandoning intuitive assumptions about agency, intention, and causation.

Because minimally counterintuitive concepts are memorable and easily transmitted, they may possess a cultural advantage over both entirely ordinary and radically incomprehensible ideas (Boyer, 2001). Barrett (2012) argues that religious beliefs often succeed because they fit naturally with existing cognitive tendencies rather than requiring extensive cognitive effort to maintain.

Norenzayan (2013) further suggests that religious systems gained additional cultural stability when they became embedded within social institutions that promoted cooperation, moral regulation, and group cohesion. In this view, religious ideas persist not only because they resonate with individual cognition but also because they serve important social functions. Atran (2002) similarly argues that religious beliefs occupy a unique position at the intersection of evolved cognitive tendencies and cultural transmission processes.

From the perspective of existential anxiety, minimally counterintuitive beliefs may be especially effective because they transform uncertainty into narratives that are both psychologically compelling and culturally shareable. They allow individuals to interpret suffering, mortality, and adversity through frameworks that appear meaningful rather than arbitrary.

### 4.3 Afterlife and continuity Bias

Beliefs concerning life after death, rebirth, ancestral existence, and spiritual continuity appear with remarkable frequency across cultures and historical periods. One possible explanation is that human cognition may be naturally inclined toward assumptions of psychological continuity beyond physical death.

Bering (2011) argues that many individuals intuitively attribute continuing mental states to deceased persons even when they consciously endorse biological death. People may find it relatively easy to imagine a dead person still knowing, seeing, feeling, or existing in some form despite recognising that bodily functions have ceased. This tendency suggests that concepts of psychological continuity may emerge naturally from ordinary cognitive processes.

Research on intuitive dualism provides further support for this perspective. Bloom (2004) proposed that humans readily distinguish between mind and body, making it easier to imagine mental existence continuing beyond physical death. Such intuitions appear early in development and may contribute to the widespread prevalence of afterlife beliefs across cultures.

Barrett (2012) similarly observed that children frequently demonstrate a tendency to attribute ongoing mental capacities to deceased individuals. Developmental research suggests that children often find complete psychological annihilation difficult to conceptualise, indicating that beliefs in continuity may arise from intuitive cognitive processes rather than solely from formal religious instruction.

These findings do not establish the truth or falsity of afterlife beliefs. Rather, they suggest that human cognition may be structured in ways that make concepts of continuation, rebirth, or spiritual persistence psychologically plausible and emotionally appealing. From the perspective of existential anxiety, such beliefs reduce the threat posed by mortality by transforming death from a final endpoint into a transition within a larger narrative of continuity.

### 4.4 Developmental encoding: childhood internalisation, authority, and attachment

Although cognitive predispositions may create receptivity to religious and existential beliefs, specific meaning systems are acquired within developmental and relational contexts. Childhood represents a particularly important period because young children are highly dependent upon caregivers for survival, protection, and explanations of the world.

Attachment theory provides a useful framework for understanding this process. Bowlby (1969, 1973, 1980) proposed that children develop internal working models of themselves, others, and the world through repeated interactions with caregivers. Ainsworth et al. (1978) further demonstrated that patterns of attachment influence how children experience security, trust, and emotional regulation. During early development, caregivers function not only as sources of physical protection but also as authoritative interpreters of reality.

Because children possess limited capacity to independently evaluate complex metaphysical or religious claims, beliefs transmitted by trusted authority figures may become deeply internalised. Barrett (2012) argues that children display a natural openness to religious explanations, while cultural and familial environments provide the specific content that shapes those beliefs. As a result, existential narratives concerning God, karma, rebirth, divine protection, destiny, or moral order may become embedded within broader systems of identity and meaning.

Kirkpatrick (2005) and Granqvist and Kirkpatrick (2016) further suggest that religious representations may function as extensions of attachment processes. For some individuals, God or other supernatural agents may serve psychological roles analogous to attachment figures by providing security, comfort, protection, and existential reassurance. Religious beliefs may therefore become emotionally encoded through the same relational systems that regulate safety and trust.

When meaning systems are repeatedly reinforced through emotionally significant relationships, community practices, rituals, and cultural narratives, they may eventually be experienced not as beliefs that were learned but as self-evident truths. This process helps explain why existential and religious convictions often feel deeply intuitive and resistant to change.

Taken together, the evidence reviewed in this section suggests that humans may be biologically and developmentally predisposed to seek structured explanations rather than purely random accounts of significant life events. Cognitive tendencies toward agency detection, pattern recognition, psychological continuity, attachment, and authority-based learning create fertile conditions for the development and transmission of meaning systems that help regulate existential anxiety. These predispositions do not determine specific beliefs, but they may help explain why moral, religious, and cosmic explanations of suffering emerge so consistently across cultures and historical periods.

## 5 Religion, moral causation, and existential regulation

Religion has historically served as one of humanity’s most powerful responses to existential anxiety. Becker (1973) argued that culture itself functions as a symbolic defence against the terror of mortality. Terror Management Theory (TMT) supports this view, demonstrating that reminders of death intensify attachment to cultural worldviews that provide meaning, order, and continuity (Pyszczynski et al., 2015).

Across traditions, religious doctrines transform death and suffering into intelligible narratives. They offer moral structure, symbolic continuity, and answers to otherwise intolerable questions. In this way, religion may reduce the psychological impact of uncertainty by embedding individual experience within a broader interpretive framework. However, religion is psychologically ambivalent: it may buffer existential anxiety, but it may also intensify it when interpreted in rigid or punitive ways.

### 5.1 Moral causation systems: sin and karma

Many religious traditions resist randomness by moralising suffering. In Christian frameworks, suffering may be linked to sin, divine judgment, or providential purpose. In Hindu and Buddhist traditions, karma provides a moral causal structure across lifetimes. These systems reduce existential uncertainty by suggesting that suffering is not arbitrary but meaningful within a moral order.

Although both sin-based and karmic traditions provide explanations for suffering, they do so through different moral logics. Sin is frequently associated with transgression, guilt, and accountability before a divine authority, whereas karma typically refers to causal continuity across actions and consequences. Karmic interpretations may involve responsibility without necessarily implying blame, punishment, or moral condemnation. The common element is not guilt but the transformation of apparently random events into intelligible forms of order.

However, this interpretive structure may also impose psychological burden. When suffering is understood as morally caused, individuals may experience self-blame, guilt, or fear of judgment. Thus, moral causation both resolves uncertainty and introduces new forms of existential distress.

### 5.2 Rebirth and continuity

Popular Buddhist and related South Asian belief systems often interpret suffering within a framework of rebirth and karmic continuity. Although classical Buddhism rejects the notion of an enduring self, it preserves causal continuity across lifetimes.

From a psychological perspective, this framework allows suffering to be understood as part of an ongoing process rather than as isolated or random events. It reduces existential discontinuity and provides a narrative that extends beyond a single lifetime, thereby mitigating the distress associated with finality and randomness.

### 5.3 Adaptive functions of religion

For many individuals, religious belief offers hope, emotional comfort, moral orientation, community belonging, and a framework through which suffering can be endured without despair. Spiritual practices such as prayer, ritual, meditation, confession, and communal worship may reduce loneliness, increase perceived social support, and foster acceptance in the face of illness, aging, and death (Koenig, 2012; Pargament, 1997). Religion may also function as an attachment-based system, providing a sense of security and relational stability during periods of vulnerability (Granqvist and Kirkpatrick, 2016).

In this sense, religion may act as a powerful regulator of existential anxiety by transforming vulnerability into connectedness, uncertainty into trust, and suffering into bearable meaning. Empirical research suggests that positive religious coping—characterised by trust, meaning, and spiritual support—is associated with improved psychological adjustment, whereas negative religious coping may increase distress (Pargament et al., 1998).

The present argument is that religion and spirituality may serve a variety of psychological functions. Their effects depend not simply on belief content, but on how beliefs are interpreted, internalised, and enacted. Under some conditions, religious meaning systems may provide comfort, resilience, and existential security; under others, they may contribute to guilt, fear, or psychological distress.

### 5.4 Religious scrupulosity and moral anxiety

Religious meaning-making is not uniformly protective. When beliefs are rigid, punitive, or moralised in ways that intensify guilt and fear, religion may amplify rather than reduce existential distress.

An extreme form of this process is *religious scrupulosity*, often conceptualised as a subtype of obsessive-compulsive disorder (Abramowitz and Jacoby, 2014). Common features include obsessive fear of sinning, compulsive prayer or confession, constant moral self-monitoring, inability to feel forgiven, and chronic fear of divine punishment.

Scrupulosity illustrates how attempts to regulate existential uncertainty through moral frameworks may paradoxically sustain chronic threat activation and psychological distress.

Religion, therefore, functions as both a *buffer against existential uncertainty and a potential amplifier of moral anxiety*. This bivalent potential provides an important foundation for the Randomness Intolerance Hypothesis developed in the following section, which proposes that individuals gravitate toward interpretive systems that reduce uncertainty while generating distinct psychological costs.

## 6 The randomness intolerance hypothesis

### 6.1 Randomness intolerance hypothesis

This paper advances the *Randomness Intolerance Hypothesis,* which proposes that human beings possess limited psychological tolerance for interpreting major life events as random. When individuals encounter significant suffering—such as illness, bereavement, betrayal, or the threat of abandonment—they experience not only emotional distress but also cognitive disequilibrium arising from a disruption of expected coherence and predictability.

Perceiving such events as random challenges fundamental psychological needs for meaning, order, and controllability. In response, individuals engage in meaning-making processes that transform uncertainty into structured interpretations. These processes are supported by well-documented cognitive tendencies, including agency detection, pattern recognition, and teleological reasoning (Barrett, 2012; Boyer, 2001), which orient individuals toward intentional and morally organised explanations of events.

Within this framework, interpretations of suffering tend to cluster around three primary pathways:

- Randomness pathway: Suffering is experienced as accidental, unjust, and uncontrollable. This interpretation may reduce moral self-blame but is associated with heightened helplessness, existential anxiety, and depressive affect.
- Moral causation pathway: Suffering is interpreted as the consequence of sin, karma, or personal failing. This reduces uncertainty by imposing moral structure, but may generate guilt, shame, and chronic self-monitoring.
- Cosmic purpose pathway: Suffering is understood as part of a larger plan, divine intention, or existential purpose. This interpretation may provide comfort, coherence, and existential security, but may also introduce behavioural risks, including passivity, fatalism, or reduced engagement with medical care.

These pathways are not mutually exclusive. Individuals may shift between them depending on context, developmental stage, and cultural background. The hypothesis is not intended to imply that meaning-making is inherently maladaptive; rather, it suggests that the *form* of interpretation determines its psychological and behavioural consequences.

**Figure 1:**
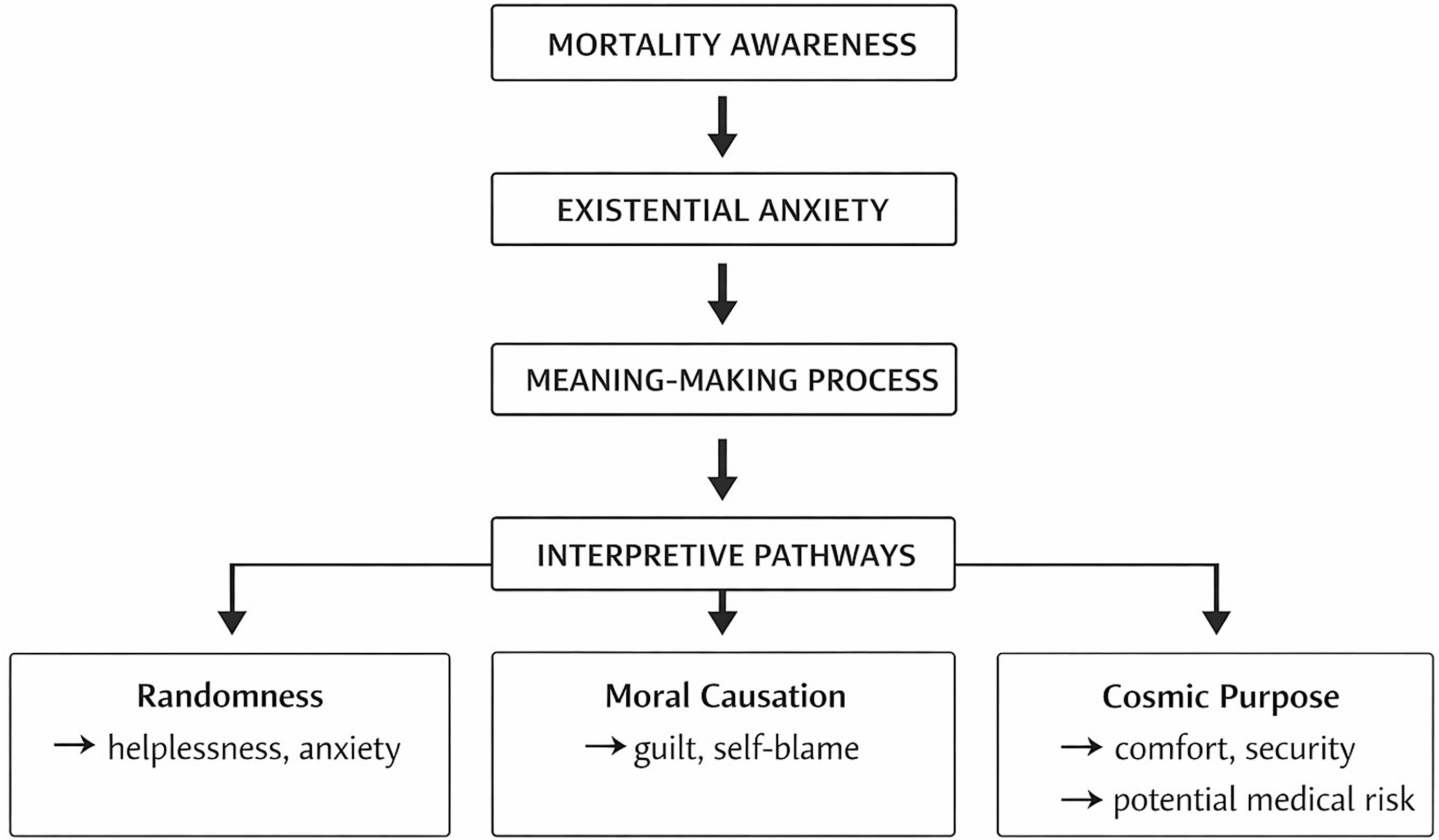
Randomness–meaning–existential anxiety model: Awareness of mortality generates existential anxiety, motivating individuals to engage in meaning-making processes. These processes produce three primary interpretive pathways: randomness, moral causation, and cosmic purpose. Each pathway reduces uncertainty while generating distinct psychological outcomes. Randomness interpretations are associated with helplessness and anxiety; moral interpretations with guilt and self-blame; and cosmic-purpose interpretations with comfort and perceived security, but may also introduce behavioural risks such as reduced engagement with medical treatment.

The Randomness Intolerance Hypothesis contributes to existing theories of existential anxiety by specifying the mechanisms through which individuals regulate uncertainty. It complements Terror Management Theory by focusing not only on the defence of cultural worldviews but on the interpretive transformation of randomness into structured meaning. In doing so, it provides a framework for understanding both adaptive and maladaptive responses to suffering. The three interpretive pathways outlined above are summarised in Figure 1.

### 6.2 Testable implications of the hypothesis

The proposed framework generates several empirically testable predictions. Individuals with higher intolerance of uncertainty (Carleton, 2016) are expected to demonstrate stronger preference for structured explanatory frameworks over randomness-based interpretations. Mortality salience manipulations may increase endorsement of moral or cosmic interpretations, consistent with Terror Management Theory. Interpretations based on moral causation may be associated with elevated guilt and anxiety, whereas randomness interpretations may correlate with helplessness and depressive affect. Finally, adaptive meaning-making frameworks that reduce perceived threat may be associated with lower physiological stress activation and improved health outcomes.

## 7 Clinical case illustrations

The following case illustrations are anonymised and partially composite vignettes derived from clinical experience. Identifying details have been altered, combined, or omitted to protect confidentiality. The cases are presented for conceptual and educational purposes and are intended to illustrate theoretical pathways rather than provide empirical evidence.

### 7.1 Case 1: mortality awareness and the distress of randomness

An 83-year-old Australian woman presented with depression, chronic pain, and existential distress. Her medical history included multiple orthopaedic surgeries, cardiovascular illness, and long-standing depressive symptoms. Progressive physical decline significantly reduced her independence, intensifying her sense of vulnerability.

Her distress escalated following the deaths of several close friends. These losses heightened mortality awareness and led her to repeatedly state that she “could not handle what is happening.” She also expressed anguish about global suffering, particularly deaths occurring in international conflicts, reflecting a sense of existential helplessness in the face of widespread and uncontrollable suffering.

Relational difficulties further compounded her distress. She described long-standing emotional pain related to her husband’s infidelity and expressed fear of abandonment. She reported wishing to die before her husband, as she felt unable to tolerate life alone.

This case illustrates existential anxiety largely unbuffered by strong religious or moral explanatory systems. Suffering—both personal and global—was experienced as arbitrary and unjust. From a psychological perspective, increased mortality salience (Greenberg et al., 1997), declining physical autonomy, empathic distress, and attachment insecurity likely interacted to amplify existential anxiety. This case exemplifies the *randomness pathway.*

#### 7.1.1 Pathway representation

This case illustrates the randomness pathway proposed in the Randomness Intolerance Hypothesis. Mortality awareness was heightened through declining health, bereavement, and increasing physical vulnerability. In the absence of a strong moral or religious explanatory framework, suffering was experienced largely as arbitrary, uncontrollable, and unjust. The resulting interpretation contributed to feelings of helplessness, anxiety, abandonment, and existential distress. The case demonstrates how difficulty tolerating randomness may amplify psychological suffering when adversity cannot be integrated into a coherent meaning system.

### 7.2 Case 2: karma and moral causation

A 79-year-old woman of South Asian background presented with depression and chronic illness following decades of hardship. Her husband had left the family when the children were young, and she raised them under significant financial and emotional strain before migrating to Australia.

She interpreted her suffering through a religious framework of karma and rebirth, believing her hardships were consequences of wrongdoing in a previous life. Her primary therapeutic request was for past-life regression to identify the moral cause of her suffering, and she showed limited interest in interventions focused on present-life circumstances.

This case illustrates how religious belief systems provide moral coherence in the face of adversity. Karmic interpretations transformed random misfortune into moral causation, thereby reducing existential uncertainty. However, this framework also appeared to reinforce self-blame and moral burden, illustrating the psychological cost of moralised meaning-making. This case exemplifies the *moral causation pathway*.

#### 7.2.1 Pathway representation

This case illustrates the moral causation pathway. Rather than viewing hardship as random misfortune, suffering was interpreted through a framework of karmic continuity and moral consequence. This interpretation reduced existential uncertainty by providing a coherent explanation for adversity and preserving a sense of causal order. However, the same explanatory framework also appeared to reinforce self-blame and moral burden. The case demonstrates how moral interpretations may simultaneously provide meaning and contribute to psychological distress.

### 7.3 Case 3: divine Providence and existential security

A 70-year-old man with a strong Catholic faith presented with multiple chronic illnesses, including cardiovascular disease and blood cancer. Religious practice was central to his life. At times, he demonstrated reluctance toward certain medical recommendations and occasionally discontinued prescribed treatment, stating that his life was ultimately “in God’s hands.”

When his adult son was admitted to hospital following a serious self-harm episode that was considered life-threatening, the family organised daily prayer gatherings. Following the son’s recovery, the client interpreted the outcome as evidence of divine intervention. This interpretation appeared to restore emotional stability by situating the crisis within divine providence.

From a psychological perspective, religious belief may function as an attachment-based source of existential security (Granqvist and Kirkpatrick, 2016). However, reliance on divine control also influenced health behaviour, illustrating how religious meaning-making may simultaneously provide comfort and introduce risk. This case exemplifies the *cosmic purpose pathway.*

#### 7.3.1 Pathway representation

This case illustrates the cosmic purpose pathway. Serious illness and the threat of loss were interpreted within a broader framework of divine providence and intentional order. The client’s belief that events unfolded according to God’s plan appeared to provide emotional comfort, existential security, and psychological stability during a period of crisis. At the same time, reliance on divine control occasionally influenced treatment adherence, highlighting how interpretations grounded in cosmic purpose may both buffer anxiety and introduce behavioural risk. The case demonstrates how existential uncertainty can be regulated through beliefs that situate suffering within a larger purposeful narrative.

Taken together, these cases illustrate the three interpretive pathways proposed in the Randomness Intolerance Hypothesis: randomness, moral causation, and cosmic purpose.

## 8 The meaning → biology → health pathway

A central proposition of this paper is that existential anxiety is not only a psychological phenomenon but also a *biologically consequential state.* The ways in which individuals interpret suffering, uncertainty, and mortality may influence physiological regulation and, over time, contribute to health or disease. This section proposes a conceptual pathway through which meaning-making processes translate into biological outcomes, referred to here as the *Meaning → Biology → Health pathway*.

### 8.1 Conceptual model

**Figure 2:**
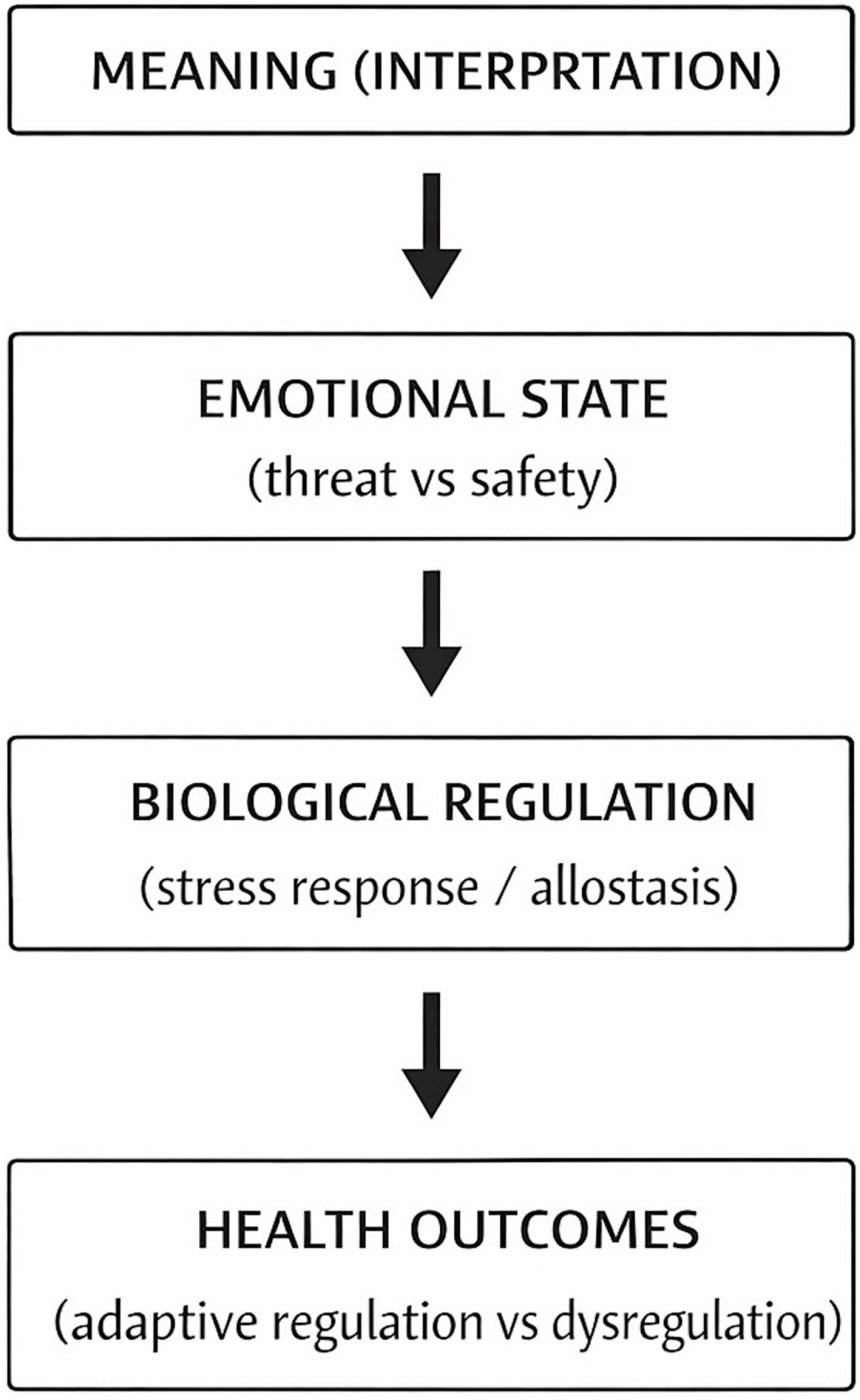
Meaning, biology & health pathway: This model illustrates how interpretive meaning influences emotional and physiological processes. Interpretations associated with threat (e.g., helplessness, guilt, fear) may activate stress responses and contribute to physiological dysregulation, whereas interpretations associated with safety and coherence may support adaptive regulation and improved health outcomes.

At the cognitive level, human beings continuously interpret internal and external events through meaning-making processes. Experiences such as illness, loss, or uncertainty are not processed as neutral stimuli; rather, they are appraised in terms of threat, safety, fairness, and personal significance. When such experiences are interpreted within frameworks of unresolved uncertainty, randomness, or perceived moral threat, they may generate persistent psychological stress. Conversely, interpretations that provide coherence, safety, or adaptive meaning may reduce perceived threat and promote emotional regulation. This relationship is illustrated in Figure 2 (*Meaning, Biology & Health Pathway*).

### 8.2 Biological mechanisms

These cognitive appraisals are closely linked to emotional states. Interpretations characterised by helplessness, fear, or self-blame are associated with chronic anxiety and heightened vigilance. In contrast, interpretations grounded in acceptance, agency, or compassionate understanding may foster emotional stability.

Importantly, these psychological states are not confined to subjective experience but are associated with measurable biological processes. Research in stress physiology has shown that chronic activation of the stress response—particularly the hypothalamic–pituitary–adrenal (HPA) axis and the autonomic nervous system—can lead to dysregulation across multiple bodily systems (McEwen, 1998, 2007). Prolonged elevation of stress hormones such as cortisol has been linked to immune dysfunction, inflammation, metabolic disturbance, and increased vulnerability to chronic disease. The concept of *allostatic load* captures the cumulative physiological burden imposed by repeated or sustained stress activation.

Psychoneuroimmunology further suggests that psychological states and immune processes are bidirectionally related (Ader, 2001). Studies on placebo and meaning responses likewise indicate that expectations and interpretations can exert direct physiological effects (Benedetti, 2014).

Empirical research supports the link between psychological meaning, stress regulation, and health outcomes. Chronic psychological stress has been associated with increased allostatic load, immune dysregulation, inflammation, and heightened risk for cardiovascular disease, metabolic disorders, and depression (McEwen, 1998, 2007; Sapolsky, 2004). Conversely, studies on religion and health suggest that individuals who engage in adaptive forms of spiritual coping often demonstrate lower levels of stress, improved psychological well-being, and, in some cases, better physical health outcomes (Koenig, 2012; Pargament et al., 1998). These findings support the view that interpretive frameworks—particularly those that reduce perceived threat and enhance coherence—may influence biological regulation through stress-mediated pathways.

At the same time, maladaptive meaning-making—such as chronic guilt, fear of punishment, or perceived lack of control—has been linked to heightened anxiety and sustained physiological activation. This suggests that the health effects of meaning-making are not determined by belief content alone, but by how those beliefs are experienced psychologically and enacted behaviourally.

### 8.3 Interpretation matters

Within this framework, existential anxiety may be understood as a persistent source of stress activation when individuals are unable to resolve or tolerate fundamental uncertainties related to death, suffering, or meaning. Interpretations of life events as random, uncontrollable, or morally threatening may sustain a state of anticipatory vigilance. When meaning-making results in chronic fear, guilt, helplessness, or perceived lack of control, it may perpetuate rather than resolve stress.

However, not all forms of meaning-making are equally adaptive. Psychological adjustment depends not only on whether an explanation provides meaning but also on whether it facilitates effective engagement with reality. Adaptive meaning-making allows individuals to acknowledge suffering while maintaining agency, flexibility, and the capacity for constructive action. In contrast, maladaptive meaning-making may reduce uncertainty at the cost of increased fear, self-blame, avoidance, or behavioural disengagement.

From a psychodynamic perspective, healthy adaptation involves balancing meaning with the reality principle—the capacity to respond to actual circumstances rather than relying exclusively on defensive interpretations. Meaning systems may therefore serve different psychological functions. Some promote acceptance, resilience, problem-solving, and repair, whereas others may reinforce helplessness, fatalism, or chronic threat perception.

Constructive meaning-making may also facilitate processes of repair and sublimation. Experiences of suffering can sometimes be transformed into caregiving, creativity, spiritual growth, community contribution, or renewed purpose. In such cases, existential challenges become integrated into broader narratives of development and adaptation rather than remaining sources of chronic distress.

Conversely, interpretations characterised by persistent self-condemnation, divine punishment, catastrophic expectation, or perceived moral failure may maintain heightened physiological arousal and contribute to long-term dysregulation. The critical issue is therefore not whether individuals construct meaning, but how that meaning influences emotional regulation, behaviour, and engagement with reality.

The central proposition of the present model is that the body responds not only to events themselves but also to the meanings attributed to those events. Interpretations shape emotional responses, behavioural choices, and physiological regulation, thereby influencing health trajectories over time.

### 8.4 Integration with existential anxiety

The Meaning → Biology → Health pathway can be integrated with the three interpretive pathways proposed in the Randomness Intolerance Hypothesis.

Interpretations emphasizing randomness may contribute to helplessness, uncertainty, and chronic anticipatory stress when individuals are unable to tolerate perceived unpredictability. Interpretations centred on moral causation may reduce uncertainty but can also generate shame, guilt, self-blame, and heightened self-monitoring. Interpretations grounded in cosmic purpose or existential meaning may provide psychological comfort and coherence, although their effects depend on how beliefs are enacted in practice.

Importantly, these pathways are not proposed as deterministic. The same belief system may function adaptively for one individual and maladaptively for another depending on context, flexibility, emotional regulation, and behavioural consequences. Meaning-making should therefore be understood as a dynamic regulatory process rather than a fixed psychological outcome.

From a biological perspective, chronic activation of stress responses is associated with increased allostatic load, dysregulation of the hypothalamic–pituitary–adrenal (HPA) axis, autonomic imbalance, immune dysfunction, and elevated inflammatory activity (McEwen, 1998, 2007). Over time, these processes have been linked to increased risk for cardiovascular disease, metabolic disorders, depression, chronic pain, and reduced overall quality of life (Sapolsky, 2004).

Conversely, interpretations that reduce perceived threat and promote emotional regulation may contribute to lower physiological stress activation and improved long-term functioning. Emerging research in psychoneuroimmunology suggests that psychological states influence immune regulation and inflammatory processes, providing a plausible mechanism through which meaning-making may affect health outcomes (Ader, 2001).

The Meaning → Biology → Health pathway therefore extends existential psychology beyond questions of subjective meaning alone. It proposes that existential interpretations become embodied through emotional, behavioural, and physiological processes that may influence health across the lifespan. In this sense, meaning is not merely a cognitive construct but a potential regulator of biological adaptation and long-term well-being.

## 9 Social amplification and buffering of meaning

Meaning is not constructed in isolation. It is shaped and stabilised within relational and cultural environments. Family systems, intimate relationships, religious communities, and broader cultural narratives all influence how suffering is interpreted.

Anxious, critical, or dysregulated social environments may amplify existential distress by reinforcing threat interpretations, helplessness, or moral fear. In contrast, supportive and regulated environments may buffer distress by promoting safety, coherence, and emotional containment. Religious communities may function in either way: as sources of belonging and stability, or as amplifiers of guilt and punitive moral surveillance.

This social dimension is clinically important because the meaning of suffering is often co-constructed. Individuals do not merely hold private beliefs; they inhabit shared interpretive worlds. These worlds can either intensify distress or foster resilience.

## 10 Clinical implications

### 10.1 Therapy focus: function rather than truth

Existential distress is often embedded within interpretive frameworks rather than presenting as isolated symptoms. Psychotherapy should therefore attend to how beliefs function psychologically rather than attempting to resolve metaphysical questions. The relevant clinical issue is not whether a particular worldview is objectively correct, but whether it increases fear, guilt, rigidity, and physiological stress, or whether it supports coherence, agency, compassion, and adaptive functioning.

From an attachment perspective, existential beliefs are often embedded within broader relational histories. Early experiences with caregivers contribute to internal working models concerning safety, trust, vulnerability, and control (Bowlby, 1969, 1973, 1980). Consequently, existential anxieties may be shaped not only by abstract beliefs about death or suffering but also by formative relational experiences. Clinical exploration of meaning systems may therefore benefit from examining how existential interpretations are connected to attachment patterns, significant relationships, and experiences of security or threat.

### 10.2 Interventions

Therapeutic work may involve increasing tolerance for uncertainty, reconstructing meaning in more flexible and compassionate ways, and integrating grief, bodily decline, and relational vulnerability. Clinicians may help clients move from threat-based interpretations toward greater acceptance, agency, and emotional complexity. In religious contexts, the aim is not to challenge faith itself but to help clients distinguish supportive spiritual meaning from fear-based or punitive interpretations.

Interventions may also focus on relational processes through which meaning is transmitted and reinforced. Families, cultural groups, religious communities, and close relationships often function as environments that either amplify or buffer existential anxiety. Therapeutic exploration of these relational contexts may help clients recognise how beliefs about suffering, morality, illness, and mortality have been acquired, maintained, or emotionally reinforced over time. In some cases, therapy may involve fostering alternative narratives that promote resilience, self-compassion, and adaptive engagement with uncertainty.

### 10.3 Health implications

If existential meaning-making influences stress physiology, psychotherapy may have significance not only for emotional distress but also for quality of life and physical health. Chronic existential fear, unresolved guilt, persistent helplessness, and threat-based interpretations may contribute to prolonged activation of stress-response systems, increased allostatic load, and associated health risks. Conversely, meaning systems that promote emotional regulation, agency, social connectedness, and adaptive coping may contribute to improved psychological and physiological functioning.

The therapeutic task therefore extends beyond symptom reduction. By helping individuals develop interpretive frameworks that do not perpetuate chronic threat activation, psychotherapy may influence downstream biological processes associated with stress regulation. Although the present model remains conceptual, it suggests that interventions targeting existential meaning-making may have implications for psychological well-being, quality of life, health behaviour, and long-term health outcomes. Future research may usefully examine whether changes in existential interpretation are associated with measurable improvements in physiological regulation and health over time.

## 11 Discussion

This paper has proposed an integrative framework linking existential anxiety, cognitive predispositions, religious meaning systems, clinical experience, and biological regulation. At its theoretical core is the *Randomness Intolerance Hypothesis,* which posits that human beings possess limited psychological tolerance for interpreting major life events as random, and therefore tend to construct moral, cosmic, or intentional explanations to restore coherence.

This framework extends existing models of existential anxiety, including Terror Management Theory, by emphasizing not only the defensive function of cultural worldviews but also the specific interpretive pathways through which individuals transform uncertainty into meaning. It further complements biopsychosocial perspectives by highlighting meaning-making as a central regulatory process linking cognition, emotion, and physiology.

The paper has also argued that these interpretive processes have consequences beyond subjective experience. Through the *Meaning → Biology → Health pathway,* existential interpretations shape emotional states, stress physiology, and long-term health outcomes. Meaning-making is therefore not merely a cognitive or philosophical activity but an embodied process with measurable biological implications.

Importantly, the analysis highlights the psychological ambivalence of religious and spiritual systems. Moderate or compassionate forms of spirituality may support emotional regulation, social connectedness, and quality of life, whereas rigid or fear-based interpretations may contribute to chronic anxiety, guilt, and physiological stress activation. This dual role underscores the importance of examining not the presence of belief, but its functional impact on the individual.

The evolutionary and cognitive science of religion provides a foundation for understanding why many individuals are predisposed toward intentional and moral explanations of suffering. Clinical case illustrations demonstrate that these interpretive systems may provide coherence and comfort, but may also generate helplessness, self-blame, or behavioural risk, particularly when they reduce engagement with adaptive coping or medical care. These processes are further shaped by social environments, which may amplify or buffer existential distress.

Taken together, the present framework suggests that human beings are not only *meaning-making beings* but *meaning-embodying biological systems,* whose interpretations of existence are translated into patterns of emotional regulation, physiological activation, and lived health outcomes.

## 12 Limitations and future research

This paper is conceptual and illustrative in nature. The clinical cases are intended to illuminate interpretive patterns rather than establish causal claims. Future research is needed to empirically examine how intolerance of uncertainty, religiosity, mortality salience, and cognitive biases such as pattern detection interact to shape explanatory styles of suffering across diverse cultural contexts.

Further work could examine whether the three interpretive pathways proposed here predict different emotional, physiological, and health-related outcomes. Longitudinal studies may be especially useful in examining whether certain forms of meaning-making buffer allostatic load while others contribute to chronic dysregulation. Quantitative research might measure intolerance of uncertainty, religiosity, moral rigidity, perceived randomness, and health outcomes simultaneously.

Future studies could empirically examine relationships between intolerance of uncertainty, religiosity, mortality salience, and health outcomes across cultural contexts.

## 13 Conclusion

Human beings live with an enduring existential tension: the need to find meaning in a world that often appears uncertain, unpredictable, and finite. When confronted with suffering, illness, and mortality, individuals are drawn toward interpretive frameworks that reduce uncertainty and restore psychological coherence. This paper has argued that this tendency reflects a fundamental limitation in the human capacity to tolerate randomness.

The Randomness Intolerance Hypothesis proposes that individuals navigate existential anxiety through three primary pathways—randomness, moral causation, and cosmic purpose—each of which provides partial resolution while introducing distinct psychological costs. These interpretive processes are not merely cognitive or philosophical; they are embodied, shaping emotional regulation, stress physiology, and long-term health outcomes through the *Meaning → Biology → Health pathway.*

By integrating existential psychology, the cognitive science of religion, and stress biology, this paper suggests that meaning-making operates as a central regulatory mechanism linking mind and body. The findings highlight the importance of distinguishing between adaptive and maladaptive forms of meaning. Interpretations that reduce perceived threat and support psychological flexibility may promote resilience and physiological regulation, whereas rigid, fear-based, or guilt-inducing interpretations may contribute to chronic distress and dysregulation.

For clinical practice, the implication is not to resolve metaphysical questions concerning the ultimate nature of suffering, but to engage with how individuals interpret their experience. Psychotherapy may therefore focus on increasing tolerance for uncertainty, fostering compassionate and flexible meaning-making, and reducing interpretations that perpetuate chronic threat activation. Understanding how individuals interpret suffering may therefore be central not only to psychological theory but also to health and clinical practice.

Ultimately, human beings are not only meaning-making creatures but *meaning-embodying organisms,* whose interpretations of existence are translated into patterns of emotional experience, biological regulation, and lived health. Understanding this relationship may provide a foundation for more integrative approaches to psychological well-being and quality of life in the face of uncertainty and mortality.

## Data Availability

No datasets were generated or analysed for this study. The manuscript presents a conceptual model and theoretical contributions supported by existing literature and anonymised clinical observations.
